# Protective Efficacy of H9N2 Avian Influenza Vaccines Inactivated by Ionizing Radiation Methods Administered by the Parenteral or Mucosal Routes

**DOI:** 10.3389/fvets.2022.916108

**Published:** 2022-07-11

**Authors:** Alessio Bortolami, Eva Mazzetto, Richard Thiga Kangethe, Viskam Wijewardana, Mario Barbato, Luca Porfiri, Silvia Maniero, Elisa Mazzacan, Jane Budai, Sabrina Marciano, Valentina Panzarin, Calogero Terregino, Francesco Bonfante, Giovanni Cattoli

**Affiliations:** ^1^Department of Comparative Biomedical Sciences, Istituto Zooprofilattico Sperimentale delle Venezie, Legnaro, Italy; ^2^Animal Production and Health Laboratory, Department of Nuclear Sciences and Applications, Joint FAO/IAEA Centre of Nuclear Techniques in Food and Agriculture, International Atomic Energy Agency (IAEA), Vienna, Austria; ^3^Department of Animal Science Food and Nutrition–DIANA, Università Cattolica del Sacro Cuore, Piacenza, Italy

**Keywords:** H9N2, vaccines, mucosal, subcutaneous, irradiated, formalin-inactivated

## Abstract

H9N2 viruses have become, over the last 20 years, one of the most diffused poultry pathogens and have reached a level of endemicity in several countries. Attempts to control the spread and reduce the circulation of H9N2 have relied mainly on vaccination in endemic countries. However, the high level of adaptation to poultry, testified by low minimum infectious doses, replication to high titers, and high transmissibility, has severely hampered the results of vaccination campaigns. Commercially available vaccines have demonstrated high efficacy in protecting against clinical disease, but variable results have also been observed in reducing the level of replication and viral shedding in domestic poultry species. Antigenic drift and increased chances of zoonotic infections are the results of incomplete protection offered by the currently available vaccines, of which the vast majority are based on formalin-inactivated whole virus antigens. In our work, we evaluated experimental vaccines based on an H9N2 virus, inactivated by irradiation treatment, in reducing viral shedding upon different challenge doses and compared their efficacy with formalin-inactivated vaccines. Moreover, we evaluated mucosal delivery of inactivated antigens as an alternative route to subcutaneous and intramuscular vaccination. The results showed complete protection and prevention of replication in subcutaneously vaccinated Specific Pathogen Free White Leghorn chickens at low-to-intermediate challenge doses but a limited reduction of shedding at a high challenge dose. Mucosally vaccinated chickens showed a more variable response to experimental infection at all tested challenge doses and the main effect of vaccination attained the reduction of infected birds in the early phase of infection. Concerning mucosal vaccination, the irradiated vaccine was the only one affording complete protection from infection at the lowest challenge dose. Vaccine formulations based on H9N2 inactivated by irradiation demonstrated a potential for better performances than vaccines based on the formalin-inactivated antigen in terms of reduction of shedding and prevention of infection.

## Introduction

Although wild waterfowl are the natural hosts of avian influenza (AI), H9N2 subtype viruses are relatively uncommon in wild birds (1). In contrast, H9N2 viruses, following their initial spread from South East Asia in the late 1990s, have become globally widespread in poultry over the last two decades, resulting in great economic losses due to their high replicative fitness in Galliformes, associated with severe drops in egg production and moderate mortality, when exacerbated by other pathogens (2, 3). In addition to the severe impact on poultry production, H9N2 viruses have also been implicated in zoonotic transmission to humans, in particular with people in direct contact with live poultry, remarking the importance of vaccination in reducing the circulation of H9N2 viruses, as an indirect measure to prevent zoonotic transmissions and reassortment events between human and AI viruses (4–6). Sustained human-to-human transmission of H9N2 viruses has not been demonstrated, but there is a piece of scientific evidence that only a few molecular changes could be needed to achieve transmissibility by respiratory droplets in humans (7). In addition to the direct involvement in zoonotic infections, H9N2 viruses have also donated the internal gene cassette to other AI viruses responsible for numerous human cases (such as highly pathogenic H5Nx viruses of the Goose/Guangdong/1996-lineage, H7N9 viruses of the Anhui/1/13-lineage, and a zoonotic H10N8 virus), often with fatal outcome (8–10).

There is no widespread consensus on the classification of H9N2 avian viruses; however, epidemiological and phylogenetic analyses of the hemagglutinin (HA) gene of H9N2 influenza viruses revealed that at least two major different lineages can be distinguished, the American and Eurasian lineage. The latter can be further divided into the BJ/94, the Y280/G9, the G1, and possibly a fourth lineage (unrelated to the previous three) found mainly in turkeys reared in Europe (11, 12).

Of the different lineages of H9N2, the G1 lineage, first detected in Hong Kong in 1997 (13) is extremely well adapted to chicken and has rapidly become endemic in poultry species after introduction into parts of Asia, the Middle East, India, Egypt, and Africa (14–17). To control the spread of the disease and to mitigate the severe economic consequences of uncontrolled virus circulation, vaccination has been applied in several endemic countries (17). In regions where these viruses are endemic, such as Asia and the Middle East, genetic and antigenic differences, have been observed within lineages circulating in specific regions (18). The effect of immune selection pressure exerted by vaccination on AI virus evolution has been previously demonstrated for H5N1 and H9N2, showing the rapid emergence of antigenic variants or selection and expansion of a variant that was present at a low prevalence when vaccination was initiated (18, 19). Antigenic drift, in a similar fashion to the antigenic drift observed in H1N1 and H3N2 seasonal human influenza strains, has also been observed in regions where vaccination against H9N2 is common (2).

Vaccines have been used to reduce clinical disease and lower the burden on the poultry industry; however, insufficient attention has been focused on the effect of vaccines on the reduction of viral shedding (2). Highly effective vaccines, able to provide sterilizing immunity, could help in reducing the evolutionary rate and the chances of recombination of H9N2 in endemic countries.

The gamma-irradiation-mediated killing of viruses was explored with little success to develop vaccines since the 1950s. However, a renewed interest in this technology has risen due to: (a) the invention of newer and safer irradiators that can deliver high doses, (b) the introduction of radio-protective compounds that can preserve antigens during irradiation, and (c) a better understanding of the immune system (20). Despite the limitations posed by the need of a radioactive source for the generation of γ-rays, irradiation offers several advantages, mainly related to the better preservation of the antigenic structure of the inactivated pathogen. It has been previously demonstrated that gamma-irradiation-inactivated influenza vaccination in mice resulted in the development of higher antibody titers and a broader spectrum of protection against antigenically different strains compared to a formalin-inactivated influenza vaccine (21–23). However, to the best of our knowledge, detailed efficacy data obtained from challenge studies evaluating irradiated avian influenza vaccines parenterally or mucosally administered to chickens or other avian species are not available in the literature.

In our work, we compared the immunogenicity and the efficacy of H9N2 experimental vaccines based on the antigens inactivated by chemical and irradiation methods, administered by mucosal or intramuscular routes in Specific Pathogen Free (SPF) White Leghorn chickens. The efficacy of the different vaccines and administration routes was measured upon challenge with different doses of an H9N2 isolate belonging to the G1 lineage, aiming to understand if the irradiation technology could improve current vaccination strategies and if the mucosal administration of the inactivated vaccines is able to elicit a protective level of immunity.

## Materials and Methods

### Virus

An AIV H9N2 isolate from the Middle East belonging to the G1 lineage (A/Chicken/Saudi Arabia/3622-31/13) was propagated and titrated in 10-day-old embryonated SPF chicken eggs (Charles Rivers) at 37°C for 72 h. Viral titrations were performed by inoculating 100 μL virus dilutions (10^−3^-10^−9^) in 10–11-day-old embryonated chicken eggs in the allantoic fluid. The inoculated eggs were incubated at 37°C and observed every 24 h to detect mortality for 7 days. Allantoic fluids harvested from the eggs were tested by the HA assay to detect viral replication, according to standard procedures (24). Allantoic fluids showing the absence of hemagglutinating activity were considered as negative for virus replication. A 50% egg infectious dose (EID_50_) was calculated according to the Reed-Muench method (25).

### Inactivation of H9N2 by Formalin and Irradiation

Inactivation by irradiation was performed at the International Atomic Energy Agency (IAEA) Laboratories in Seibersdorf, Austria, by following established protocols. The virus stock was mixed with 1M trehalose (trehalose dihydrate; Sigma) 50% V/V, aliquoted into 5 mL volume, and immediately frozen. Frozen samples incubated with dry ice were irradiated using a Model 812 Co-60 irradiator at a dose rate of 66.532 Gy/min (Foss Therapy Services, Inc., California, USA). The irradiator was regularly calibrated using an ionization chamber that also mapped the delivered dose in the location where the samples are irradiated. Initial doses of 5, 10, 15, 20, 25, 30, and 40 kGy were applied to identify the D10 value, the dose required to reduce virus load by 90% or 1 log (26). All samples were labeled with P8100 radiation indicator stickers that progressively change from yellow to purple depending on the dose applied ranging from 3 to 25 kGy (GEX Cooperation, Colorado, USA). The D10 value was used to estimate the inactivation dose. Formalin-inactivated H9N2 was prepared at the Istituto Zooprofilattico Sperimentale delle Venezie (IZSVe) following previously described protocols (27). Briefly, 0.1% V/V formalin in phosphate-buffered saline (PBS) was added to the infectious allantoic fluid and incubated at 37°C for 16 h. To compare vaccine preparations that only differed by the type of inactivation, after formalin treatment, the allantoic fluid was then mixed with 1M trehalose (trehalose dihydrate; Sigma) 50% V/V. Loss of viral infectivity was confirmed by three blind passages of treated viruses in embryonated eggs. The inactivated virus suspensions were stored at −80°C until further use.

### Transmission Electron Microscopy (TEM)

Aliquots of untreated, formalin-inactivated, and irradiated viruses were analyzed by negative staining TEM according to standard procedures for viral identification and examination. A formvar/carbon supported copper grid (Electron Microscopy Sciences Formvar/Carbon Copper Grid 200 Mesh) was placed flat on the bottom of the vial and 90 μL of samples were dispensed on the top of the grid. After high-speed centrifugation (28–30 psi or 100,000 × *g*) for 15 min (Beckman Air-Driven Ultracentrifuge Airfuge), the grid was placed on a filter paper and stained with 10 μL of 2% phosphotungstic acid (PTA) (pH 7); PTA was left on the grid for a few seconds (8–10 s). The grid was then examined under an EM 208S TEM (Philips) and virus particles were measured using the iTEM software (Olympus SIS).

### Animal Experiments

#### Bird Infectious Dose 50 (BID_50_)

BID_50_ determination was based on the methods described by Swayne and Slemons (28). Birds were challenged with serial dilutions of the selected strains. For each tested dilution, groups of five SPF White Leghorn chickens (*Gallus gallus*) 4–6 weeks old were housed in poultry isolating units (Montair, The Netherlands). All the birds in each group were infected *via* the oronasal route with 100 μL of viral suspension in PBS containing the corresponding EID_50_ dose (one dose per group). Only tracheal swabs (FLmedical, Italy) were collected daily from day 1 to day 5 post-infection (p.i.), as previous experimental results (data not shown) indicated that cloacal shedding was negligible (mean Ct values >30). The samples were then processed for the detection of the M gene by real-time RT-PCR (RRT-PCR) (29). The BID_50_ was defined using the Spearman and Kärber method (30).

#### Animal Trial 1 (H9N2 Challenge Dose: 10^6^ EID_50_/100 μL)

A total of 40 one-day-old SPF White Leghorn chickens were equally divided into five groups and housed in BSL3 poultry isolators (HM 1900, Montair Andersen BV, Kronenberg, The Netherlands). Two groups were vaccinated oculo-nasally (ON) with either the irradiated-H9N2 (ON-Irr) or the formalin-inactivated H9N2 (ON-For) antigens without adjuvants, respectively. The other two groups were vaccinated subcutaneously (SC) with either irradiated-H9N2 (SC-Irr) or formalin-inactivated H9N2 (SC-For) antigens, respectively, in a water-in-oil (W/O) 7:3 (v/v) emulsion with a commercial adjuvant for poultry (ISA71VG, Seppic). A fifth group served as a negative non-vaccinated control. All groups were vaccinated twice at 14 and 28 days of age and blood samples were taken before each vaccination. The amount of H9N2 antigen given to each bird was standardized to 128 hemagglutinating units (HAU) for each immunization. Two weeks after the second dose (i.e., 42 days of age) blood samples were taken from all the chickens and a homologous challenge was performed by the oronasal route at a dose of 10^6^ EID_50_/100 μL. Tracheal swabs were collected from all the birds on day 2, 4, and 7 p.i. to evaluate viral shedding. Fourteen days p.i. (dpi), a final blood sample was taken from all the birds to evaluate seroconversion.

#### Animal Trial 2 (H9N2 Challenge Dose: 10^3^ and 10^4^ EID_50_/100 μL)

A total of 150 one-day-old SPF White Leghorn chickens were divided into five different experimental groups. The first group was vaccinated ON with irradiated-H9N2 (ON-Irr-Adj) in a 1:1 suspension with the mucosal adjuvant IMS1313 (Seppic, France), the second group received by the ON route a formalin-inactivated H9N2 vaccine in a 1:1 suspension with IMS1313 (ON-For-Adj). The other two groups were vaccinated SC in the same way as in animal trial 1 (SC-Irr, SC-For). The amount of H9N2 antigen given to each bird was standardized to 128 HAU for each immunization. A fifth group served as negative non-vaccinated control. All the vaccinated birds received two doses of the experimental vaccines at 14 and 28 days of age and blood samples were taken before each vaccination. Two weeks after the second dose (i.e., 42 days of age) blood samples were taken, and within each group, birds were equally divided into subgroups of 15 birds each and challenged with either 10^3^ or 10^4^ EID_50_/100 μL of the homologous virus. Clinical signs were monitored daily and tracheal swabs for quantification of viral shedding were collected on day 1, 2, 3, 4, 5, 7, and 9 p.i. Fourteen days p.i., a final blood sample was taken from all the birds to evaluate seroconversion.

### Assessment of Viral Shedding by Real-Time RT-PCR

RRT-PCR targeting the M-gene was used to determine the BID_50_ and to compare viral shedding in the respiratory tract in each experimental group, in a qualitative and quantitative setup, respectively (29). Swab heads were placed in 500 μL of 1X PBS containing antibiotics and antimycotics (PBS-A) and vortexed for 30 s. Total RNA was purified from 300 μL of sample suspension using the QIAsymphony® DSP Virus/Pathogen Midi Kit on a QIAsymphony® SP instrument (Qiagen). Viral genome amplification was carried out using the QuantiTect Multiplex RT-PCR Kit (Qiagen), 300 nM of each primer, 100 nM of the probe, and 5 μL of template RNA, in a final volume of 25 μL. Each sample was tested in triplicate. Runs were performed on a CFX 96 Deep Well Real-Time PCR System, C1000 Touch (Biorad), under the following cycling conditions: 50°C for 20 min, 95°C for 15 min, followed by 40 cycles at 94°C for 45 s and 60°C for 45 s.

Ten-fold serial dilutions of strain-specific negative-sense *in vitro* transcribed RNA were processed along with each run to develop standard curves and to assess viral shedding. The limit of quantification (LoQ) of the RRT-PCR was preliminarily assessed as being 10^0.7^ genome copies.

Viral replication was plotted as the mean viral load ± SD using the Prism 9.1.2 (GraphPad). For graphical and statistical purposes, samples testing negative or with a viral load below the LoQ were given a value of 10^0.7^ copies/5 μL of total RNA.

### Serological Assays

To detect the humoral immune response of vaccination, hemagglutination inhibition (HI) assays and a commercial ELISA assay targeting the nucleoprotein (NP) of type A influenza viruses were performed on all serum samples collected during animal trials 1 and 2. HI assays were performed according to standard protocol using the homologous vaccine antigen (31). In brief, sera were serially diluted in PBS and mixed with equal volumes (25 μL) of the virus containing 4 HAU, then 25 μL of washed chicken red blood cells were added and incubated for 30 min at room temperature. HI titers were determined as reciprocals of the highest serum dilutions in which inhibition of hemagglutination was observed.

The anti-NP ELISA (ID Screen® Influenza A Nucleoprotein Indirect, IDVet, France) was performed according to the manufacturer's recommendation using positive and negative controls provided with the commercial kit.

### Statistical Analyses

The shedding dynamics from 1 to 12 dpi of the control population and those administered with formalin-inactivated and the irradiated vaccines were modeled through General Additive Model (GAM). GAM was performed as implemented in the ‘mgcv' R package, which was also used to assess the concurvity and significance of base functions, model selection was performed through the Akaike information criterion (AIC), and the model assumptions were verified through the graphical assessment of the models' residuals using the R package ‘gratia' (32–34). A GAM was fitted for each challenge dose (10^3^ − 10^4^ − 10^6^) and for each administration route (ON and SC). Shedding observations equal to zero were increased to one (from hereon: Shed01); all observations were then log-transformed. Due to the limits of detection of 10^0.7^ copies/5 μL, shedding levels presented a distinct zero-inflation. Consequently, we implemented a two-components mixture GAM where the probability of attaining value 0 (fit0) and the probabilities of the non-0 values (fit1) are modeled separately, and the coefficients from the two models are joined to return a single response model (fit). To compute a different smooth for each unique treatment while allowing for varying intercept, models fit0 and fit1 included the treatment both as a fixed factor and as a factor-smoothing parameter for DPI. The response variable for fit0 was a binary variable describing the presence/absence of measured shedding for each given observation and modeled as logistic regression with a binomial distribution of errors and logit link function. To model fit1, Shed01 was log-transformed and modeled with a Gaussian distribution of errors and log link function. The predicted values from both models were then joined as **fit=e**^log(**fit0**)**+**log(**fit1**)^. Confidence intervals at the 95% confidence level (95% CI) were inferred generating 1,000 bootstrap resampling and applying a bias-corrected CI as implemented in the “coxed” R package (35, 36).

To assess the overall shedding difference significance among treatments for each dose/route combination, general linear mixed models (GLMM) of the log-transformed Shed01 were fitted using treatment as a fixed factor, DPI, and sample ID as random variables as implemented in the “lme4” R package (37). The “emmeans” R package (38) was used to compute the estimated marginal means and the contrast among treatments; the *p*-values associated with the contrast were corrected for multiple comparisons through Honestly Significant Difference (HSD) adjustment.

## Results

### Inactivation and Preservation of Structural Integrity

The D10 dose was identified as 5.46 kGy (Supplementary Figure 1). An inactivation dose of 60 kGy was used for vaccine preparation and was estimated by adding four D10 doses to the minimum inactivation dose estimated at 35.68 kGy to ensure effective sterilization of the virus. The final dose of 60 kGy, was within the range of the SAL and determined safe for use. Around 12.5 h were taken to deliver this irradiation dose using a gamma irradiator and the sample was kept frozen all the time by refilling dry ice. Indeed, there was a slight difference in each time the inactivation took place as the Co-60 source decayed over time.

**Figure 1:**
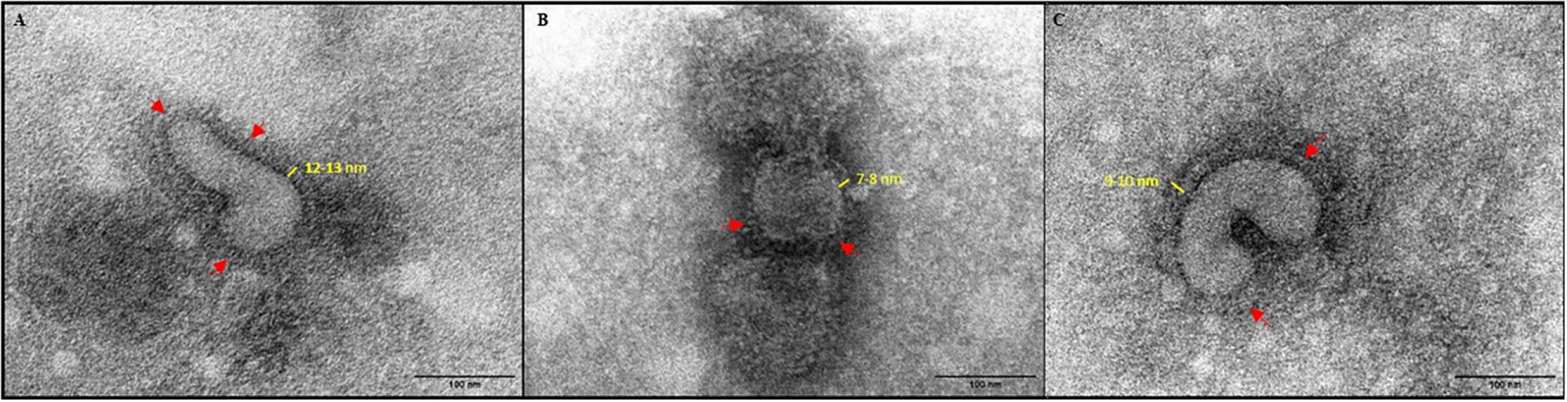
Negative stain TEM images of virus (× 180,000). **(A)** Live untreated H9N2. **(B)** formalin-inactivated H9N2; and **(C)** irradiated H9N2. Red arrows indicate viral glycoprotein spikes.

Inactivation and safety of formalin-treated and irradiated H9N2 used in the experimental vaccines were confirmed by three blind passages in 10-day-old embryonated eggs. Additionally, no loss in HA titer was observed irrespective of the inactivation method used. Upon Transmission electron microscopy (TEM) examination (Figure 1), both formalin and γ-irradiation treatments showed no effect on the integrity of viral particles and normal morphology was preserved. However, after examination of several viral particles, formalin-fixed virions exhibit shorter and less easily detectable projections, representing the immunogenic glycoproteins on their surface than the irradiated viral particles (Figures 1B,C, respectively).

### BID_50_ of A/Chicken/Saudi Arabia/3622-31/13

To infer the BID_50_ of the challenge virus, we performed multiple infection experiments at different challenge doses (10^3−6^ EID_50_/100 μL) in poultry isolators units. Following the challenge, tracheal swabs were collected daily and RRT-PCR tests were run to identify infected birds. The results of the challenge are shown in Table 1 and the BID_50_ was determined as 10^3.5^ EID_50_/100μL.

**Table 1:** BID_50_ determination in 6-weeks-old White Leghorn SPF chickens, each infection experiment was performed by oronasal installation of 100 μL of infectious allantoic fluid diluted in PBS to five (*n* = 5) SPF chickens in different isolator units.

**Challenge dose**	**Positive chickens**	**Negative chickens**	**% of infected** **(Mean Ct of infected chickens at day 1 p.i.)**
**10** ^**3**^	2	3	40 (33.2)
**10** ^**4**^	3	2	60 (25.2)
**10** ^**5**^	5	0	100 (21.4)
**10** ^**6**^	5	0	100 (23.1)

*The challenge dose is expressed as EID_50_/100 μL*.

### Vaccination by the Subcutaneous (SC) Route

Formalin-inactivated vaccines represent the most common type of traditional vaccine available for AI. Despite the extensive knowledge of the protection offered by inactivated vaccines when administered SC, control of H9N2 infection is difficult under field conditions and most of the countries in which vaccination is applied are still endemic to H9N2. In our work, we aimed to compare the protective efficacy of formalin-inactivated and irradiation-inactivated H9N2 experimental vaccines against different challenge doses of a homologous virus to the vaccine antigen.

The serological analysis showed that both formulations, when administrated SC, were able to produce high-antibody titers in immunized birds before challenge (i.e., Geometric Mean Titer (GMT) >10 log_2_ in all of the immunized groups) according to both HI and NP-ELISA tests. Higher mean HI titers were observed in birds immunized in trial 2 compared to trial 1, possibly due to improved vaccine preparation methods (extended emulsion time, higher shearing speed, and preparation performed on ice), which were adjusted following discussion with the manufacturer. Nonetheless, in SC-For and SC-Irr groups challenged with the same dose, no significant difference was observed in the GMTs.

**Figure 2:**
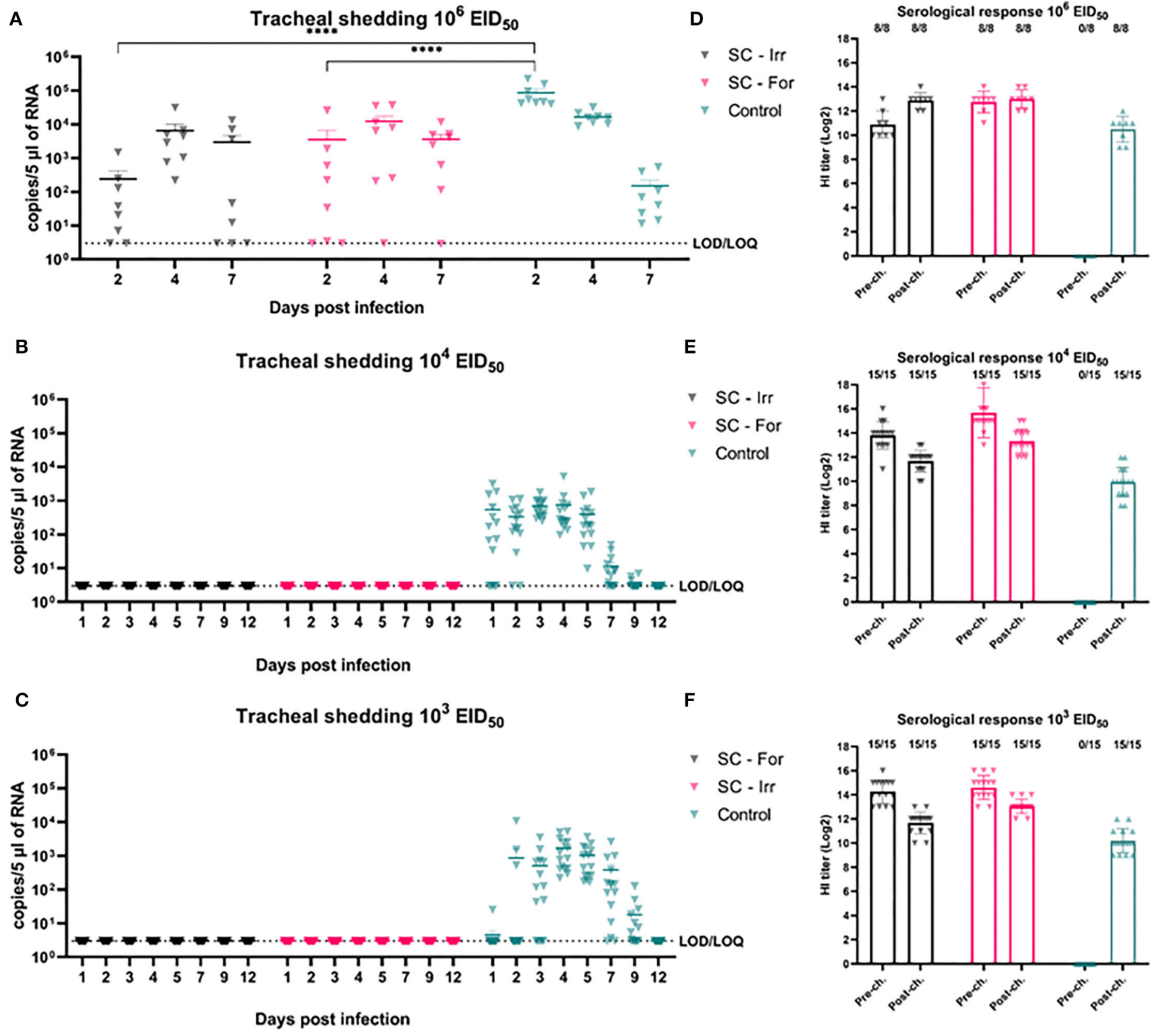
Effects of subcutaneous (SC) vaccination upon infection at different H9N2 challenge doses. **(A–C)** qRRT-PCR results of tracheal swabs collected from all the challenged birds. **(D–F)** Serological test results of blood samplings performed before and after challenge. The numbers above bars indicate a number of NP-ELISA positive birds out of tested birds.

**Figure 3:**
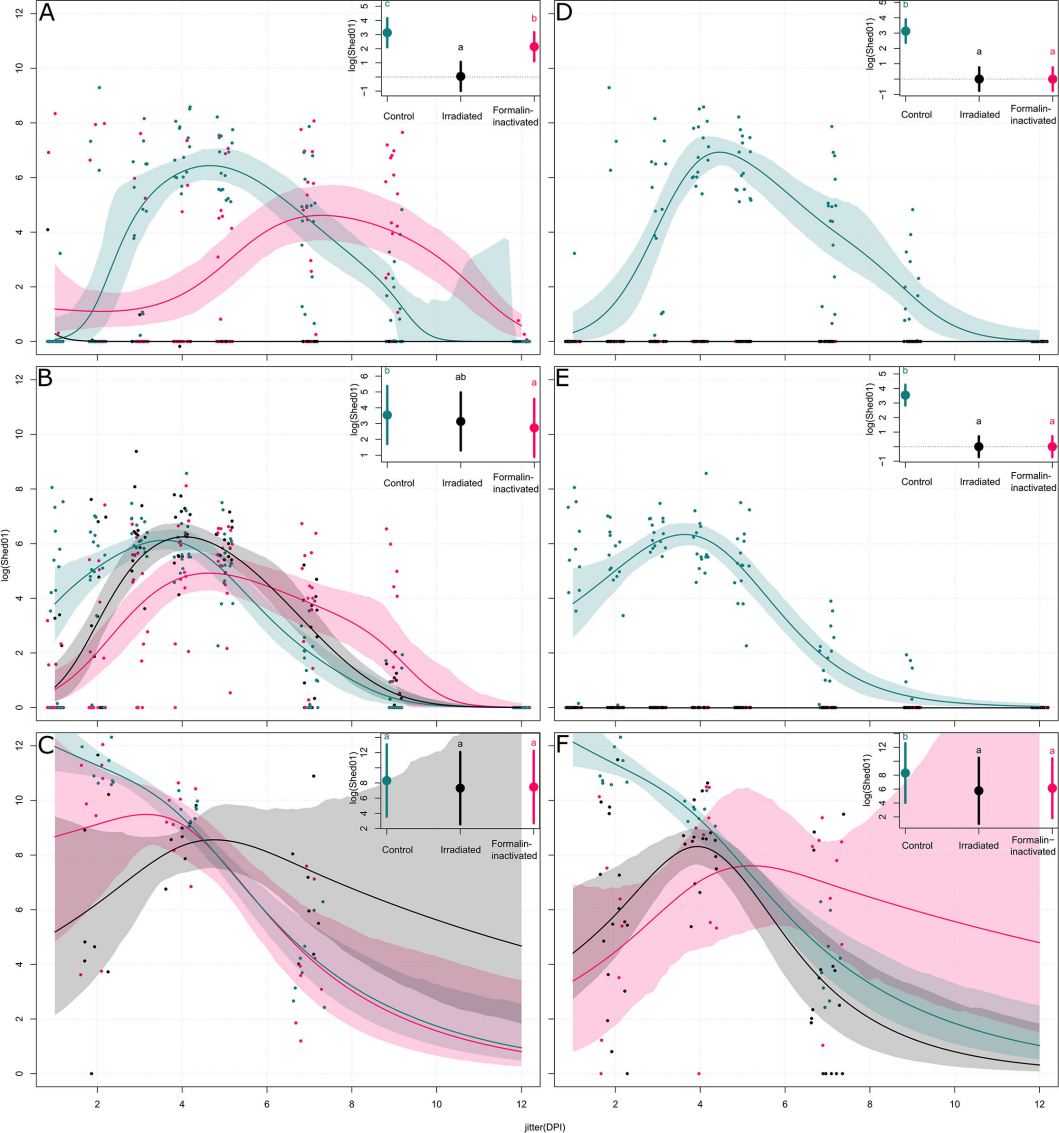
Models of the shedding levels of irradiated and formalin-inactivated vaccines and a non-vaccinated control for 12 days post infection (DPI) for three challenge doses (10^3^, 10^4^, and 10^6^ EID_50_). For each group, the dots represent the measured observations, the solid line represents the fitted model, and the shaded contour shape represents the 95% confidence intervals of the fitted model. For each plot, the inset compares the overall effect of the group on the shedding, with the solid circle representing the effect value and the vertical bars the 95% confidence interval. Panels **(A–C)** show models of shedding in ON vaccinated birds upon challenge with 10^3^, 10^4^, and 10^6^ EID_50_ of H9N2, respectively; panels **(D–F)** show models of shedding in SC vaccinated birds upon challenge with 10^3^, 10^4^, and 10^6^ EID_50_ of H9N2, respectively.

In animal trial 1, we challenged chickens with a 10^6^ EID_50_ dose (i.e., 10^2.5^ times greater than the BID_50_) of the H9N2 isolate. Quantitative RRT-PCR was performed on tracheal swabs collected on days 2, 4, and 7 p.i. showed only partial virological protection in birds, irrespective of the type of inactivation method. However, on day 2 p.i., viral shedding was significantly lower in SC-Irr (10^2.38^ ± 10^2.70^ copies/5 μL) and SC-For (10^3.54^ ± 10^3.94^ copies/5 μL) groups than in the control chickens (10^4.94^ ± 10^4.83^ copies/5 μL), but differences between the two vaccinated groups were not statistically significant. In both vaccinated groups, 75% (6/8) of birds resulted to be positive on day 2 p.i. (Supplementary Table 1), as opposed to 100% (8/8) in the control group. In a comparison with the control group, we observed lower mean viral loads for both groups at day 4 p.i. but higher loads at day 7 p.i. (Figure 2A). Nonetheless, on day 7 p.i., 3/8 and 1/8 chickens resulted negative in the SC-Irr and SC-For groups, respectively, while in the control groups all birds were found positive. A GLMM statistical approach for the analysis of the viral shedding aggregated data (Figure 3) over 12 dpi. was applied to model infection dynamics. The analysis based on the RRT-PCR data showed that the effect of SC vaccination upon challenge with 10^6^ EID_50_ mainly affects the initial phases of the infection by reducing the number of infected animals upon challenge for both the vaccines, albeit the effect of vaccination did not reach statistical significance (Figure 3F, inset).

We then evaluated the protective efficacy at lower challenge doses (10^3^ and 10^4^ EID_50_), below and above the BID_50_, to better discriminate differences in the ability of these vaccines to prevent an infection. Shedding results showed complete prevention of infection in all challenged birds, resulting in 100% efficacy of both the experimental vaccines in preventing infection (Figures 2B,C). Following the challenge, none of the vaccinated birds recorded an increase in the HI titers (Figures 2D–F).

### Vaccination by the Mucosal (ON) Route

Mucosal vaccination of the upper respiratory tract in poultry is an attractive alternative to SC vaccination due to the potential advantages offered by mass administration and the capacity of mucosal vaccines to elicit mucosal immunity at the site of entry of respiratory viruses. To assess differences in the protective efficacy between irradiated and formalin-inactivated H9N2 antigens administered by the mucosal route, we performed three challenges, including doses of 10^3^, 10^4^, and 10^6^ EID_50_.

Serological analyses showed variable HI titers before the challenge in all vaccinated birds and no significant differences were observed in terms of HI GMT between formalin inactivated and irradiated vaccinated groups (3.93 log_2_ and 4.71 log_2_, respectively). Interestingly, NP-ELISA results were clearly distinguishable. The NP-ELISA performed on sera collected before the challenge gave negative results in the irradiated vaccinated groups, while in the formalin inactivated groups few (3/38, 7.9%) chickens seroconverted. After the challenge, the NP-ELISA showed seroconversion in the infected chickens.

**Figure 4:**
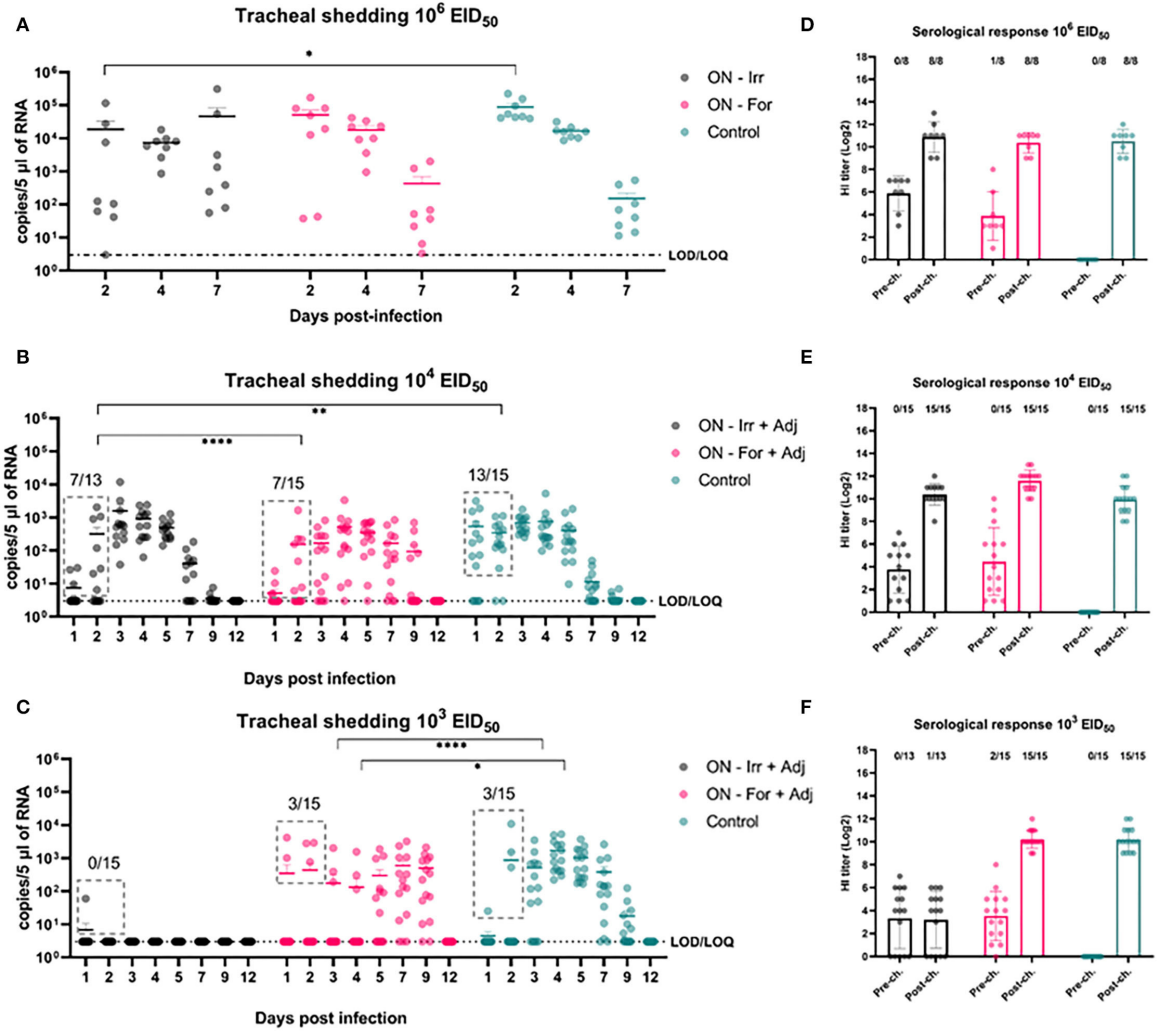
Effects of mucosal (oculo-nasal, ON) vaccination upon infection at different H9N2 challenge doses. **(A–C)** qRRT-PCR results of tracheal swabs collected from all the challenged birds. **(D–F)** Serological test results before and after challenge. The numbers above bars indicates the number of NP-ELISA positive birds out of tested birds.

As shown in Figure 4, at the highest challenge dose 8/8 of the unvaccinated birds infected directly from challenge and high viral titers (10^4.94^ ± 10^4.83^ copies/5 μL) were detected in tracheal swabs, as early as 2 dpi. In contrast, vaccinated birds showed more heterogeneous shedding titers at the early stages of infection. In particular, a significant reduction in mean viral load was observed in the ON-Irr group compared to controls on day 2 p.i. (Figure 4A). Moreover, in the ON-Irr group, 7/8 challenged birds resulted positive at 2 dpi. However, due to the high transmissibility of the H9N2 virus in chickens, all birds were infected at 4 dpi in all the groups. The GLMM model built on aggregated shedding data shown in Figure 3C failed to detect statistically significant differences in terms of overall shedding between groups.

At lower challenge doses, the effect of mucosal vaccination on the prevention of infection was evident, recording fewer positive birds compared to the control group during the first 2 days after the challenge. Upon inoculation with 10^4^ EID_50_, the percentage of positive birds in the ON-Irr-Adj, the ON-For-Adj and the control groups ranged between 23.1% (3/13)−53.8% (7/13), 26.7% (4/15)−46.65 (7/15) and 66.7% (10/15)−86.6% (13/15), respectively (Supplementary Table 1). Mean viral loads were significantly higher on day 3 p.i. for the ON-Irr-Adj group, while no statistical difference was recorded for samples at other time points. After day 3, all birds resulted positive at least for two consecutive days (Supplementary Figure 2), recording shedding peaks at days 3, 4, and 4 p.i. for ON-Irr-Adj, ON-For-Adj, and controls, respectively.

Upon challenge with 10^3^ EID_50_, the percentage of positive birds in the ON-Irr-Adj, the ON-For-Adj and the control groups ranged between 6.7% (1/15)−0.0% (0/13), 13.3% (2/15)−20.0% (3/15) and 6.7% (1/15)−20.0% (3/15), respectively (Supplementary Table 1). In the ON-Irr-Adj group, we observed a transient positivity in one bird at day 1 p.i., while no other animal resulted positive throughout the 12 days. In the ON-For-Adj group the same three infected birds that were positive on day 2 p.i. remained the only animals shedding virus up to day 4 p.i., while on day 5 p.i., five additional subjects resulted positive. In the control group, in addition to the three directly infected birds observed on day 2 p.i. six positive subjects were recorded on day 3 p.i. (Supplementary Figure 2). In the ON-For-Adj and the control groups, all birds resulted infected for at least two consecutive days, recording shedding peaks at days 7 and 4 p.i., respectively. HI titers increased after the challenge in all the groups except for the ON-Irr-Adj group challenged with a dose of 10^3^ EID_50_. In this group, the only chicken that transiently shed low viral loads on day 1 p.i. resulted in NP-ELISA positive at 14 dpi.

## Discussion

Vaccination to prevent H9N2 AIV infection in poultry has been used extensively since the late 1990s first in China and then in regions that became endemic following the global spread of these poultry-adapted viruses (18, 39, 40). Vaccination programs have relied heavily on traditional vaccines based on oil-emulsified, inactivated whole AIVs (39, 41) to reduce the severe economic consequences of infection. Inactivation of infectious allantoic fluid for the preparation of vaccines destined for the poultry market is usually achieved by formaldehyde treatment (31), which represents an effective well-established method. However, formalin treatment has been demonstrated to affect viral antigenicity by HA polymerization (27) and by reducing the host-immune response to the inoculated antigen. Moreover, formalin, at commercial vaccine concentration levels, has been demonstrated to negatively affect production performances in laying hens by causing degeneration in combs, follicles, oviduct, and uterus and lower estradiol levels (42).

In our study, we demonstrated that irradiation is a valid alternative to formalin for the inactivation viruses, as previously demonstrated for other human influenza strains (22) or other pathogenic viruses, such as rotavirus (43), Venezuelan Equine Encephalitis virus (44), and Ebola virus (45). Complete inactivation of an infectious allantoic fluid with a titer of 10^8^ EID_50_, demonstrated by blind passaging in embryonated chicken eggs, was confirmed for irradiation doses higher than 40 kGy. Visualization of inactivated viral particles by TEM imaging suggested that formalin treatment affected more than γ-irradiation of the viral structure by reducing the height of surface immunogenic glycoproteins and by causing a more clustered appearance of H9N2 envelope projections, probably as a result of the cross-linking effect of the formalin treatment (46). The superiority of γ-irradiation to other chemical inactivation methods in the preservation of antigenic structures has been previously demonstrated and is due to the selective damaging effect of irradiation on the RNA genetic material, and the limited impact that irradiation has on proteins if frozen conditions are maintained during the inactivation process (47–49). In our work, to minimize the deleterious effects of γ-irradiation, we also used trehalose as a radio-protectant to preserve the antigenic epitopes during the irradiation process. Trehalose is a well-known cryo-protectant that stabilizes proteins and has been shown to protect DNA during radiation (50). Indeed, trehalose has been used widely in viral vaccine formulations to achieve stabilization of the antigens (51, 52). Moreover, being a sugar it can also aid in increasing the viscosity leading to increased attachment and prolonged presence of vaccine antigens in the mucosae. On the other hand, it should be noted that the addition of trehalose and performing irradiation under frozen conditions could have increased the gamma irradiation dose needed for the inactivation of the virus because of the protective effects exerted both on viral proteins and the viral genome (53). Not surprisingly other groups who used gamma irradiation to inactivate influenza virus at room temperature and without trehalose achieved complete inactivation at 16 KGy (54).

When administered SC, no significant differences were recorded in terms of immunogenicity between the two vaccines. However, HI titers were higher than previously reported for similar antigen concentrations (50, 55), possibly as the result of either optimal vaccine preparation and administration (e.g., type of adjuvant) or due to the presence of trehalose in vaccine batches, whose activity on the modulation of host immunity is well-documented (56). Excellent results of ISA71VG as an adjuvant for AIV vaccine preparation have also been previously demonstrated in a study performed by Lone and colleagues (57) who compared 10 different commercial and experimental adjuvants for use in chickens, identifying ISA71 VG to perform best in terms of clinical protection and reduction of viral shedding in experimentally infected SPF chickens. We believe that the addition of trehalose to mineral oil adjuvants in vaccine formulations should be further investigated considering its viscosity and adjuvant properties.

When birds were challenged with 10^6^ EID_50_, SC vaccinated groups recorded a significant reduction of shedding (10^1.40^-10^2.56^ fold reduction) in the trachea and a reduced number of positive birds in the early phase of the study, on day 2 p.i. Nonetheless, such reduction did not affect the overall number of birds becoming infected in the vaccinated groups, while it delayed the peak of shedding to day 4 p.i. Interestingly, although mean loads on day 7 p.i. were higher in both vaccinated groups than in the control group, the irradiated and the formalinated vaccines afforded viral clearance in 3/8 and 1/8 animals, respectively, as opposed to the control group in which all birds were still actively shedding the virus. At lower challenges, both vaccines provided sterilizing immunity according to both virological and serological results, achieving a result rarely described in the literature for AI (58). The superior adaptation of H9N2 viruses of the G1 and Y280 lineages to the respiratory epithelia of Galliformes reduces shedding a challenging task for the current inactivated vaccines. In our setting, we proved that even extremely high homologous HI titers could not prevent infection and transmission of infection upon high challenges, reminding us that the traditional approach to AI vaccination *via* IM/SC immunizations with inactivated vaccines might be sufficient to prevent H9N2 infection, only in the presence of moderate loads. High HI titers are known to efficiently curb the commercial impact of H9N2 disease (59, 60), but in our setting proved inefficient in reducing the circulation of a virus characterized by low BID_50_ and high sustained shedding profiles. Altogether, the irradiated antigen performed better than the formalin-inactivated, although differences did not reach statistical significance.

When ON vaccinated groups were challenged with 10^6^ EID_50_, no significant differences were recorded in terms of cumulative shedding in comparison with the control group. However, a significant reduction in shedding was observed in the ON-Irr-adj group at 2 dpi leading to a substantial delay in the shedding dynamic, albeit failing to effectively reduce the circulation of the virus in the flock. This scenario closely replicated what was observed with the SC vaccination. To effectively immunize animals *via* the mucosal route, an inactivated antigen-based vaccine must overcome tissue-specific challenges, in fact, mucosal surfaces display a broad tolerance to antigens and harbor several barriers to the delivery of antigens, such as cilia (mechanical), mucus (chemical), and proteolytic enzymes (biochemical) (61). In an attempt to improve antigen delivery at the level of the mucosa, in the second animal trial, we added to the vaccine formulation IMS1313, an adjuvant with immunostimulatory activity developed for mucosal administration of live vaccines, which showed promising results also with inactivated AIV vaccines (62). When ON immunized chickens were infected with lower doses of H9N2, a stronger reduction in the percentage of positive birds became evident, especially during the first 2 days after the challenge. Both the irradiated and formalinated vaccines dramatically reduced the infection rate against the 10^4^ EID_50_ dose. Although overall cumulative shedding did not differ between these two vaccines, the duration was shorter for the ON-Irr-adj group. On the other hand, at the lowest challenge of 10^3^ EID_50_, the irradiated vaccine was the only one affording a complete protection from infection in the entire flock, while the formalinated vaccine did not prevent the infection of 3/15 birds, similarly to what was observed in the control group. Once again, we observed a delayed replication of the virus with high viral loads recorded around 7–9 days from challenge. As we expected that challenges with doses of 10^3−4^ EID_50_ would lead to primary infection in about 40–60% of birds according to BID_50_ for this virus, we assume that the observed higher percentages of RRT-PCR positive birds recorded from 3 dpi in both unvaccinated groups were the result of the secondary spread between primarily infected and primarily non-infected birds.

For this reason, although we could not differentiate primary from secondary infections, we speculate that the number of positive birds identified during the first 2 days after the challenge, might be largely attributable to primarily infected birds. In light of this, the better performance of the irradiated vaccine during the early phases of the lowest challenge with 10^3^ EID_50_ suggests the possibility that irradiated antigens administrated *via* the mucosal route might have reduced the primary attack rate more effectively than formalinated ones.

This might depend on the higher avidity/affinity of secretory IgA (S-IgA) mounted against a better preserved antigenic structure inactivated through irradiation (63). A limitation of our study is that we did not assess the IgA levels at the humoral and the mucosal level. Nonetheless, although protection offered by mucosal vaccination cannot be thoroughly measured by the HI assay, lower HI titers in the ON groups correlated with poorer performances when compared to the SC vaccinated animals. The addition of a mucosal adjuvant for live vaccines did not increase mean HI titers, possibly as the result of the lower viscosity of the formulation. Further studies are necessary to test novel mucoadhesive adjuvants (e.g., nanoparticles) and adjuvants targeting receptors of mucosal immune cells that could increase the permanence of inactivated antigens at the mucosal level, and thus increasing interaction with mucosal immune cells and the stimulation of the immune system at a crucial anatomical site for the establishment of infection.

Altogether, our results indicate that vaccination with an antigen inactivated by gamma irradiation achieves excellent results in terms of prevention of infection against low-to-intermediate H9N2 challenges if the vaccine is administrated SC. Moreover, irradiation of the antigen resulted in a shorter duration of shedding when compared to the traditional formalin-inactivated antigen. Additional experimental evidence on the efficacy of irradiated antigens in protecting against H9N2 and other AI subtypes is necessary to confirm our observations and understand whether this method is either comparable or superior to others currently used in vaccine manufacturing. This inactivation method might represent an alternative to the traditional formalin-based approach, especially in light of the recent advancements replacing radioactive material with the safer and cheaper low-energy electron irradiation technology. Although ON vaccination with an inactivated antigen only partially reduced replication against a high challenge, the performance against a low-to-moderate challenge with a highly infectious strain of H9N2 proved the potential of this innovative delivery route, in particular when the antigen was inactivated by irradiation. Undoubtedly, vaccination by spray or mixing in drinking water can stimulate the mucosae of the upper respiratory and digestive tracts, and is also less expensive and more easily applicable in emergency situations than the SC vaccination and can be designed for periodic boosters. Interestingly, mucosal vaccination with the irradiated H9N2 antigen revealed a complete lack of seroconversion against the structural NP, offering a possible Differentiating Infected from Vaccinated (DIVA) approach that would simply rely on existing commercial anti-NP ELISA assays.

A significant reduction of environmental contamination is one of the secondary goals of vaccination campaigns in endemic countries where human exposure to zoonotic H9N2 viruses is of concern. Noticeable achievements in this sense have been recorded after the deployment of nationwide immunization against H5/H7 HPAI and LPAI viruses in China, with a dramatic drop in the number of H7N9 cases (64, 65). Improving the ability of H9N2 vaccines in reducing shedding, environmental contamination, and increasing the resilience of animals to infection is not only a priority to safeguard poultry production and the access to low-cost proteins in lower-income countries but also a desirable objective from a public health perspective. Mucosal vaccination with either live-vectored vaccines or inactivated antigens might offer the chance to achieve these goals.

## Data Availability Statement

The original contributions presented in the study are included in the article/Supplementary Material, further inquiries can be directed to the corresponding author/s.

## Ethics Statement

The animal study was reviewed and approved by Istituto Zooprofilattico Sperimentale delle Venezie Ethics Committee and the Italian Ministry of Health.

## Author Contributions

CT, FB, and GC conceived and designed the study and supervised the study. AB, EM, VW, RK, and FB performed experiments. JB, SMan, EM, LP, and SMar performed laboratory analyses. MB performed statistical analyses of the generated datasets. AB and FB wrote the manuscript. VW, CT, VP, and FB assisted in the experimental design and preparation of the manuscript. All authors contributed to the article and approved the submitted version.

## Funding

The present work was developed within the framework of the Coordinated Research Project (N° D32033), Irradiation of Transboundary Animal Disease (TAD) Pathogens as Vaccines and Immune Inducers, funded by IAEA.

## Conflict of Interest

The authors declare that the research was conducted in the absence of any commercial or financial relationships that could be construed as a potential conflict of interest.

## Publisher's Note

All claims expressed in this article are solely those of the authors and do not necessarily represent those of their affiliated organizations, or those of the publisher, the editors and the reviewers. Any product that may be evaluated in this article, or claim that may be made by its manufacturer, is not guaranteed or endorsed by the publisher.
