# Supplementary material for: Protective Efficacy of H9N2 Avian Influenza Vaccines Inactivated by Ionizing Radiation Methods Administered by the Parenteral or Mucosal Routes
Source: Front Vet Sci. 2022 Jul 11;9:916108. doi: 10.3389/fvets.2022.916108 (PMC9309530; doi:10.3389/fvets.2022.916108)
Supplement: Supplementary file 1 [file Data_Sheet_1.docx]

Supplementary Material


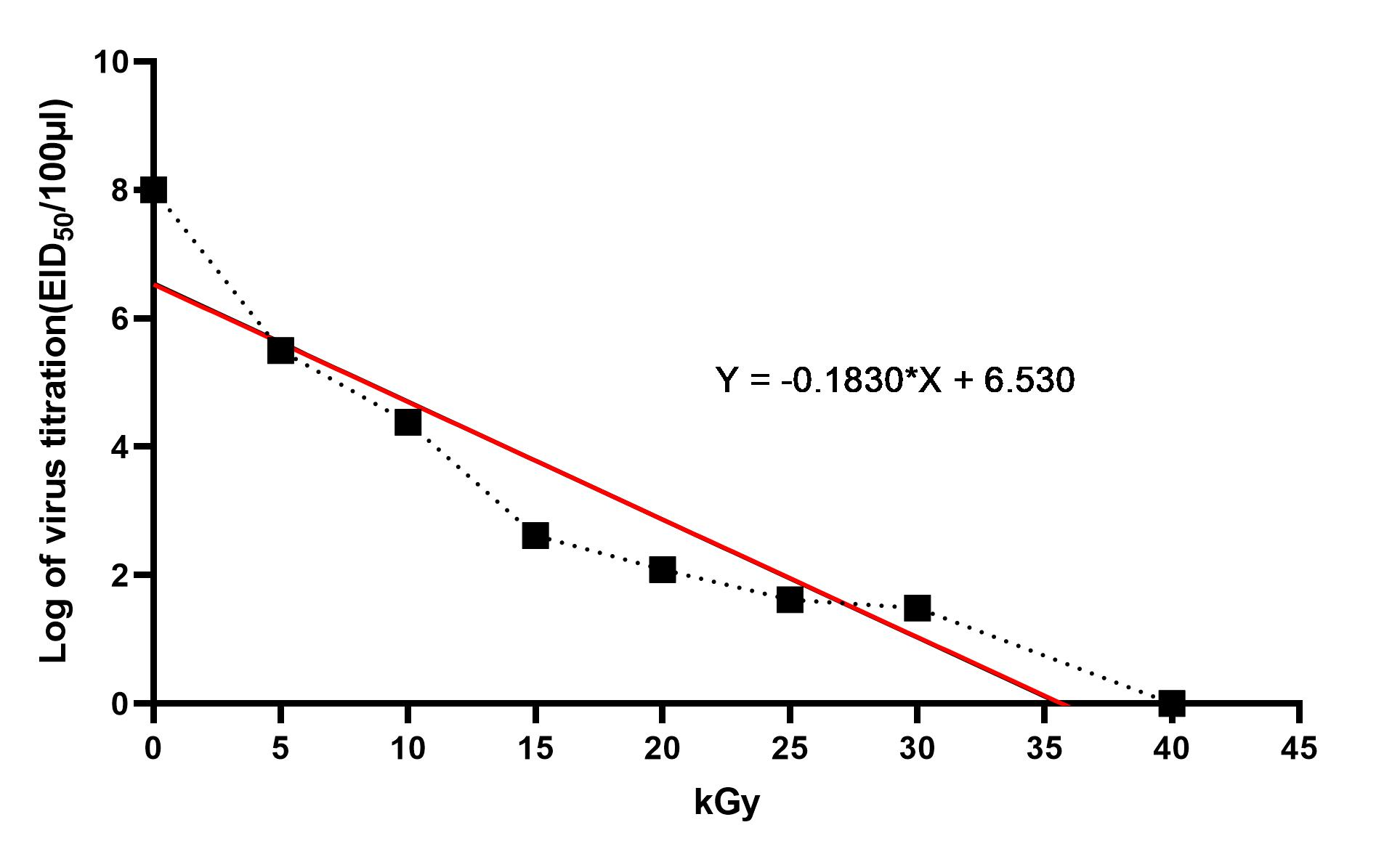


**Supplementary Figure 1.** Infectivity of H9N2 virus suspensions irradiated at doses of 5, 10, 15, 20, 25, 30, 40 kGys.

**Supplementary Table 1.** Number of chickens in each experimental group with virus titers above the Limit of Detection (LoD) at tracheal level as detected by qRRT-PCR at each sampled time point.

| **Challenge dose** | **Experimental group** | **Number of chickens shedding virus above LoD (%)** | | | | | | | |
| --- | --- | --- | --- | --- | --- | --- | --- | --- | --- |
|  |  | **1 DPI** | **2 DPI** | **3 DPI** | **4 DPI** | **5 DPI** | **7 DPI** | **9 DPI** | **12 DPI** |
| **10^3^ EID_50_** | ON-Irr-Adj | 1 /15 (6.7) | 0/15  (0) | 0/15  (0) | 0/15  (0) | 0/15  (0) | 0/15  (0) | 0/15  (0) | 0/15  (0) |
|  | ON-For-Adj | 2/15 (13.3) | 3/15  (20) | 3/15 (20) | 3/15 (20) | 8/15  (53.3) | 12/15  (80) | 14/15  (93.3) | 0/15  (0) |
|  | SC-Irr | 0/15  (0) | 0/15  (0) | 0/15  (0) | 0/15  (0) | 0/15  (0) | 0/15  (0) | 0/15  (0) | 0/15  (0) |
|  | SC-For | 0/15  (0) | 0/15  (0) | 0/15  (0) | 0/15  (0) | 0/15  (0) | 0/15  (0) | 0/15  (0) | 0/15  (0) |
|  | Control | 1  (6.7) | 3  (20) | 9  (60) | 15  (100) | 15  (100) | 13  (86.7) | 8  (53.3) | 0/15  (0) |
| **10^4^ EID_50_** | ON-Irr-Adj | 3/13  (23.1) | 7/13  (53.9) | 13/13  (100) | 13/13  (100) | 13/13  (100) | 8/13  (61.5) | 2/13  (15.4) | 0/15  (0) |
|  | ON-For-Adj | 4/15  (26.7) | 7/15  (46.7) | 11/15  (73.3) | 14/15  (93.3) | 14/15  (93.3) | 12/15  (80) | 6/15  (40) | 0/15  (0) |
|  | SC-Irr | 0/15  (0) | 0/15  (0) | 0/15  (0) | 0/15  (0) | 0/15  (0) | 0/15  (0) | 0/15  (0) | 0/15  (0) |
|  | SC-For | 0/15  (0) | 0/15  (0) | 0/15  (0) | 0/15  (0) | 0/15  (0) | 0/15  (0) | 0/15  (0) | 0/15  (0) |
|  | Control | 10/15  (66.7) | 13/15  (86.7) | 15/15  (100) | 15/15  (100) | 15/15  (100) | 10/15  (66.7) | 2/15  (13.3) | 0/15  (0) |
| **10^6^ EID_50_** | ON-Irr | - | 7/8  (87.5) | - | 8/8  (100) | - | 8/8  (100) | - | - |
|  | ON-For | - | 8/8  (100) | - | 8/8  (100) | - | 7/8  (87.5) | - | - |
|  | SC-Irr | - | 6/8  (75) | - | 8/8  (100) | - | 5/8  (62.5) | - | - |
|  | SC-For | - | 5/8  (62.5) | - | 7/8  (87.5) | - | 7/8  (87.5) | - | - |
|  | Control | - | 8/8  (100) | - | 8/8  (100) | - | 8/8  (100) | - | - |


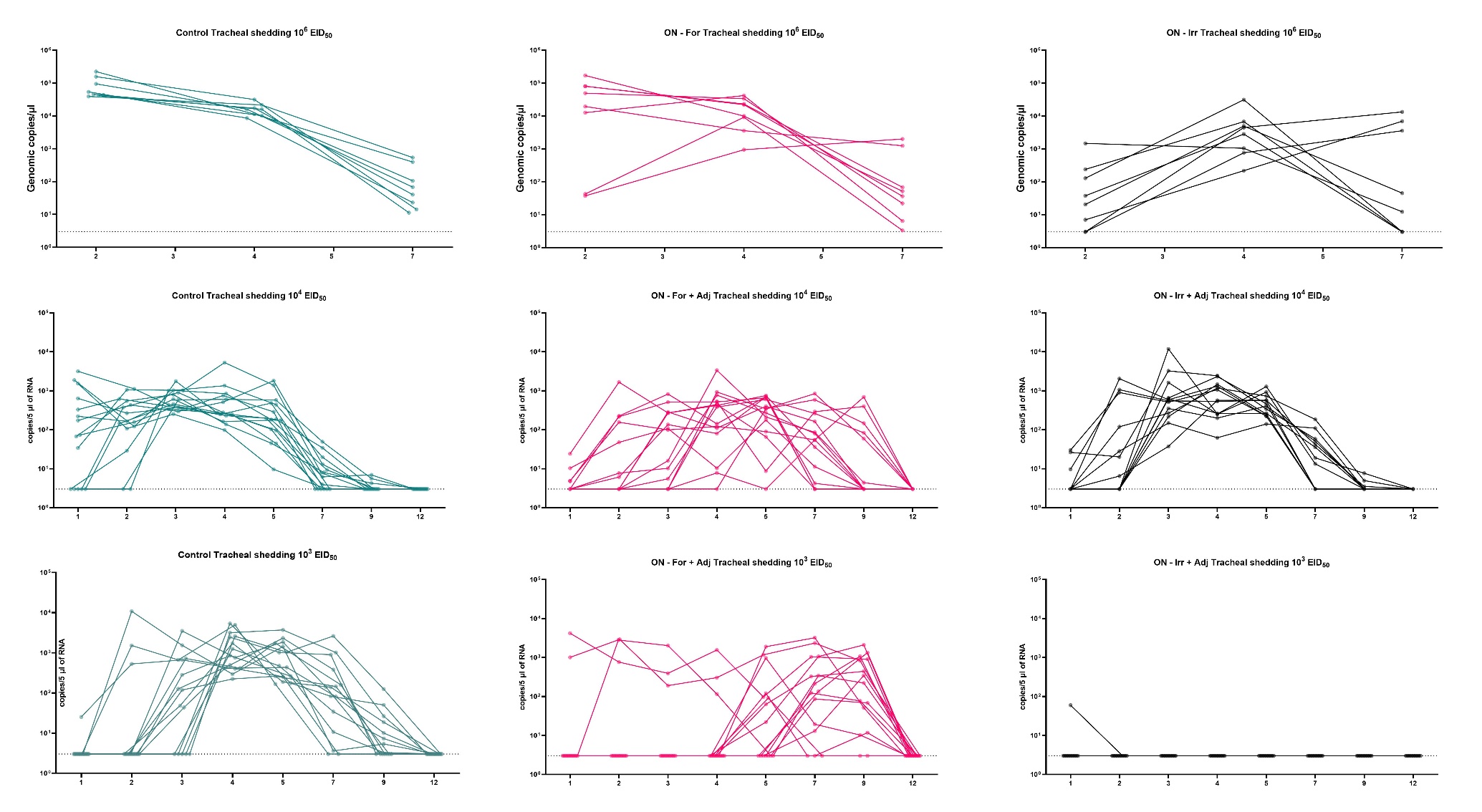


**Supplementary Figure 2.** Individual shedding curves of chicken vaccinated ON and of control groups at the three challenge doses (10^6^, 10^4^, 10^3^ EID_50_)
